# Yoga in Correctional Settings: A Randomized Controlled Study

**DOI:** 10.3389/fpsyt.2017.00204

**Published:** 2017-10-16

**Authors:** Nóra Kerekes, Cecilia Fielding, Susanne Apelqvist

**Affiliations:** ^1^Department of Health Sciences, University West, Trollhättan, Sweden; ^2^R&E, Swedish Prison and Probation Services, Norrköping, Sweden

**Keywords:** yoga, prison, impulsivity, attention, positive and negative affect, antisocial behavior

## Abstract

**Background:**

The effect of yoga in the reduction of depressive symptoms, anxiety, stress, anger as well as in the increased ability of behavioral control has been shown. These effects of yoga are highly relevant for prison inmates who often have poor mental health and low impulse control. While it has been shown that yoga and meditation can be effective in improving subjective well-being, mental health, and executive functioning within prison populations, only a limited number of studies have proved this, using randomized controlled settings.

**Methods:**

A total of 152 participants from nine Swedish correctional facilities were randomly assigned to a 10-week yoga group (one class a week; *N* = 77) or a control group (*N* = 75). Before and after the intervention period, participants answered questionnaires measuring stress, aggression, affective states, sleep quality, and psychological well-being and completed a computerized test measuring attention and impulsivity.

**Results:**

After the intervention period, significant improvements were found on 13 of the 16 variables within the yoga group (e.g., less perceived stress, better sleep quality, an increased psychological and emotional well-being, less aggressive, and antisocial behavior) and on two within the control group. Compared to the control group, yoga class participants reported significantly improved emotional well-being and less antisocial behavior after 10 weeks of yoga. They also showed improved performance on the computerized test that measures attention and impulse control.

**Conclusion:**

It can be concluded that the yoga practiced in Swedish correctional facilities has positive effects on inmates’ well-being and on considerable risk factors associated with recidivism, such as impulsivity and antisocial behavior. Accordingly, the results show that yoga practice can play an important part in the rehabilitation of prison inmates.

## Introduction

In general, yoga has received less scientific attention than meditation. This can be partially explained by the fact that yoga is a more complex, multifaceted intervention than meditation. The different components of yoga and the vast array of yoga disciplines have made it challenging to pin down its specific effects. Despite these difficulties, research on yoga has increased, and there are now several studies and systematic reviews that support the use of yoga in both clinical and healthy populations. Studies has shown that yoga is associated with improvements in mood and emotional well-being (1–3), anxiety and stress (4–7), and anger and verbal aggression (7, 8), as well as reduction of symptoms in patients with schizophrenia and schizoaffective disorder (9, 10), chronic treatment-resistant PTSD (11), and depressive symptoms (4, 6, 7, 12). Research has mainly focused on the health benefits of yoga, but there are also studies that has examined the effects of yoga on gene expression, brain activity, and cognition, showing that yoga practice is linked to improved immunity and stress regulation (13) better emotion regulation, cognitive control (14), improved memory (4), spatial recall (15), and sustained attention (16).

Prison populations, based on inmates’ high levels of aggressive antisocial behavior (17) and frequent psychiatric problems (18, 19), are specific targets for implementation of effective techniques, which may alleviate these factors. In a randomized controlled trial, Bilderbeck et al. (20) evaluated a yoga discipline that is almost identical to the yoga practiced in Swedish correctional facilities. Compared to participants in the control group, participants who completed a 10-week yoga course showed significant improvements in positive affect, perceived stress and psychological distress. On a cognitive-behavioral task administered at the post-intervention assessment, yoga participants performed significantly better than controls on the parts of the task that measured sustained attention and concentration. Those who attended more yoga classes, and engaged in frequent self-practice, reported greater decrease in perceived stress and negative affect than those who did not attend the classes or do self-practice (21).

In the first meta-analysis of the effects of yoga and meditation in correctional settings, it was concluded that there was sufficient evidence to suggest that yoga and meditation have promising effects for inmate populations (22). A larger effect size was demonstrated for psychological well-being (such as stress, negative affect, and self-esteem) than for behavioral functioning (such as impulsivity and substance use), suggesting that yoga and meditation have a more immediate positive effect on well-being than on behavior. Furthermore, the meta-analysis showed that interventions of longer duration and lower intensity had a slightly larger positive effect on behavioral functioning compared to interventions of shorter duration and higher intensity.

Yoga practice in Swedish correctional facilities started in 2002. It became recognized as a national assignment in 2008 and was named Krimyoga. It was developed by the Prison and Probation Services’ national yoga coordinator in cooperation with Prison Phoenix Trust in Oxford, UK. Krimyoga is based on Hatha yoga, which is a physical yoga that includes elements of relaxation and meditation.

This study explored if 10 weeks of yoga practice in correctional settings was associated with lower degrees of stress, aggression and negative affect, and higher degrees of positive affect, impulse control, sleep quality, and psychological well-being.

## Materials and Methods

### Procedure

Nine medium- and high-security correctional facilities were included in the study, including two with female inmates. With one exception, prison officers, who also held the yoga classes were in charge of participant recruitment, background data collection, and administering the pre- and post-intervention assessment. The Swedish Prison and Probation office provided yoga teacher training.

Data was collected from November 2013 to July 2015. However, data collection at the two female correctional facilities ended in January 2015, due to difficulties in recruiting female participants.

**Figure 1:**
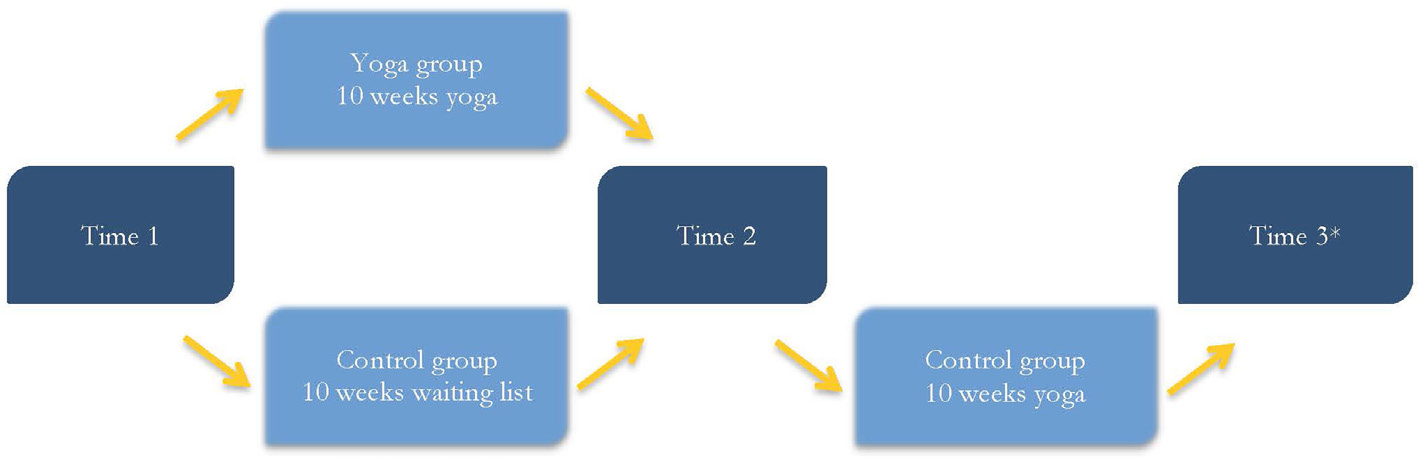
The study procedure after participants had been assigned to the yoga or control group. *Time 3 assessment was optional.

Participants were continuously included in the study. Individuals with limited or no proficiency in the Swedish or English language, and those with less than five months remaining at the correctional facility were excluded from the study. Participants completed a pre-intervention assessment (henceforth, *Time 1*), which consisted of a battery of self-report questionnaires and a computerized test, measuring attention and impulsivity. They were then randomly assigned to either yoga classes or a waiting list. During the 10-week intervention period, participants in the yoga group attended a weekly, teacher-led, 90-min yoga class. Participants in the control group were assigned to a yoga wait list for 10 weeks and asked to perform some other type of physical activity for 90 min each week. After the intervention period, participants in the yoga and control groups completed a post-intervention assessment (henceforth, *Time 2*). Participants in the control group who went on to practice yoga for 10 weeks were offered the opportunity to complete an additional post-intervention assessment (henceforth, *Time 3*). Figure 1 illustrates the study procedure after the randomization routine.

### Participants

Information about the participants’ age, offenses in their current sentence, and length of sentence was collected. Offenses were categorized into the following categories [Swedish National Council for Crime Prevention (23)]: offenses against life and health (such as homicide, assault, involuntary manslaughter); offenses against liberty and integrity (such as unlawful threats, unlawful deprivation of liberty, violation of a woman’s integrity); sexual offenses (such as rape, sexual exploitation, sexual molestation); acquisitive offenses (such as theft, robbery, shoplifting); fraud (such as fraud, extortion, fencing); and drug-related offenses (such as possession, distribution, smuggling). Offenses that did not fit into any of the above categories were labeled as other offenses (such as accounting and tax offenses, traffic violations, vandalism, weapons offenses). Some participants were convicted of multiple offenses in their current sentence, therefore were placed in more than one offense category.

#### Attrition

A total of 226 inmates (201 males; 25 females) were recruited for the study. Of these, 134 (121 males; 13 females) were randomly assigned to the yoga group and 92 (80 males; 12 females) to the control group. Seventy-four participants (68 males; 6 females) did not complete the study. Therefore, a total of 32.7% of participants were lost to follow-up. Analyses showed that participants lost to follow-up were significantly younger than those who completed the study (mean age 32.9 years compared to 35.7 years). On the pre-intervention assessment, participants lost to follow-up had significantly higher scores on measures of aggression (mean score 7.7, compared to 4.4) and antisocial behavior (mean score 4.0, compared to 2.2) than participants who remained in the study.

Reasons for attrition were categorized into the following areas: participant’s request; transfer; misconduct; did not attend all yoga classes; illness, injury, or mental health problems; missing data; and yoga class interfered with school or work. Table 1 presents attrition rate and reasons for attrition in the yoga and control groups.

**Table 1:** Attrition rates and reasons for attrition in the yoga and control groups.

	Number (%)		
	Yoga group (*n* = 134)	Control group (*n* = 92)	*p*
Attrition rate	57 (42.5)	17 (18.5)	<0.001
**Reason for attrition**			
Participant’s request	16 (28.1)	10 (58.8)	0.044
Transfer	13 (22.8)	5 (29.4)	0.79
Misconduct	6 (10.5)	0 (0)	0.39
Did not attend all yoga classes	6 (10.5)	0 (0)	0.39
Illness, injury, or mental health problems	6 (10.5)	1 (5.9)	0.98
Missing data	4 (7.0)	1 (5.9)	1.00
Yoga class interfered with school or work	4 (7.0)	0 (0)	0.69
Not specified	2 (3.5)	0 (0)	1.00

As Table 1 shows, there was a significant difference in attrition rates between the yoga and control groups (*n* = 57; 42.5% in the yoga group and *n* = 17; 18.5% in the control group). In both groups, the most common reason for attrition was at the participant’s request. However, a greater proportion of participants in the control group left the study on their own request compared to participants in the yoga group (*n* = 10; 58.8% in the control group and *n* = 16; 28.1% in the yoga group).

#### Participant Characteristics

The final sample included 152 participants (133 males; 19 females), evenly distributed between the yoga and control groups. Participants in the two groups were compared to see if they differed, despite the random assignment to groups. Table 2 describes the participants in the yoga and control groups in terms of gender, age, offenses in current sentence, length of sentence, and prison security level.

**Table 2:** Participant characteristics, including comparisons between yoga and control group participants.

	Yoga group (*n* = 77)	Control group (*n* = 75)	*p*
**Gender**, male/female	67/10 (87.0/13.0%)	66/9 (88.0/12.0%)	1.00
**Age**, *M* (min–max; SD)	36.4 (18–53; 9.4)	34.9 (20–58; 9.9)	0.20
**Offense in current sentence^a^**				
Offenses against life and health	24 (31.2%)	37 (49.3%)	0.034
Offenses against liberty and integrity	13 (16.9%)	14 (18.7%)	0.94
Sexual offenses	10 (13.0%)	13 (17.3%)	0.60
Acquisitive offenses	15 (19.5%)	8 (10.7%)	0.20
Fraud	6 (7.8%)	3 (4.0%)	0.52
Drug-related offenses	22 (28.6%)	13 (17.3%)	0.15
Other offenses	12 (15.6%)	15 (20.0%)	0.62
**Length of sentence in months,^^b^^** *M* (min–max; SD)	36.6 (5–168; 31.7)	44.8 (8–204; 41.9)	0.24
**Security level**, high/medium	17/60 (22.1/77.9%)	9/66 (12.0/88.0%)	0.15

*^a^The sum is greater than the total number of participants, due to some participants having multiple offenses in their current sentence*.

*^b^Three participants were sentenced to life imprisonment and excluded from calculation of sentence length*.

Participants in the yoga and control groups were comparable in gender distribution, age, length of sentence, and security level of holding prison. To a great extent, the two groups were also comparable in offense in current sentence. However, there was one significant difference between the two groups in offense in current sentence. A greater proportion of participants in the control group were convicted of offenses against life and health than participants in the yoga group (*n* = 37; 49.3% in the control group compared to *n* = 24; 31.2% in the yoga group).

Statistical analyses were also conducted to explore if the yoga and control groups differed in terms of scores on the Time 1 assessment (pre-intervention). There were no significant differences between the yoga and control groups on any of the variables at Time 1 (for mean scores on all variables at Time 1 and Time 2, see Table 3).

**Table 3:** Average ratings at pre- and post-intervention assessment (Time 1 and Time 2).

	Within groups								Between groups	
	Yoga group (*n* = 77)				Control group (*n* = 75)					
Variable (range of scores)	Time 1	Time 2	Average change	*p*_within_	Time 1	Time 2	Average change	*p*_within_	*d*	*p*_between_
**Perceived stress**										
**Perceived Stress Scale—14 Items (PSS-14)**										
PSS-14 total score (0–56)	25.6 (9.2)	22.4 (9.0)	−3.8 (8.3)	<0.001	27.2 (9.6)	25.7 (9.3)	−1.7 (9.0)	0.033	0.24	0.34
**Aggression**										
**Prison adjusted measure of aggression (PAMA)**										
Aggression (0–25)	4.6 (6.8)	1.8 (3.6)	−2.8 (6.3)	<0.001	4.2 (5.6)	2.8 (4.8)	−1.2 (6.0)	0.06	0.26	0.33
Self-directed aggression (0–10)	0.5 (1.3)	0.2 (0.6)	−0.4 (1.1)	0.002	0.3 (1.0)	0.3 (1.4)	−0.0 (1.6)	0.66	0.25	0.20
Antisocial behavior (0–20)	2.4 (4.1)	1.2 (2.2)	−1.3 (3.9)	0.008	2.0 (3.6)	2.0 (4.2)	0.5 (4.1)	0.80	0.45	0.046
PAMA total score (0–55)	7.5 (10.5)	3.4 (5.9)	−4.2 (9.6)	<0.001	6.4 (9.2)	5.2 (9.4)	−0.0 (9.7)	0.41	0.44	0.12
**Positive and negative affect**										
**Positive and negative affect schedule—expanded form 30 items**										
Positive activated (0–50)	30.4 (8.7)	31.7 (7.4)	1.3 (7.6)	0.18	28.6 (8.6)	30.4 (8.2)	1.8 (7.7)	0.09	0.07	0.92
Positive deactivated (0–25)	13.7 (4.4)	15.3 (4.1)	1.7 (4.1)	<0.001	13.3 (4.6)	13.7 (4.4)	0.5 (4.0)	0.44	0.29	0.027
Negative activated (0–50)	20.8 (7.9)	17.6 (6.2)	−3.1 (5.6)	<0.001	21.2 (8.0)	20.4 (8.2)	−0.9 (7.4)	0.23	0.33	0.041
Negative deactivated (0–25)	11.8 (3.4)	10.8 (3.4)	−1.1 (3.9)	0.020	12.7 (4.6)	12.3 (4.6)	−0.4 (4.1)	0.66	0.18	0.13
**Attention and impulse control**										
**Conners’ Continuous Performance Test II^a^**										
Omissions (0–100)	59.8 (50.1)	53.8 (28.0)	0.1 (31.3)	0.27	58.0 (39.1)	60.2 (30.7)	1.9 (43.9)	0.23	0.05	0.09
Commissions (0–100)	53.7 (10.9)	48.6 (11.3)	−5.5 (7.3)	<0.001	56.1 (11.3)	56.0 (11.9)	−0.3 (7.2)	0.40	0.72	<0.001
Hit reaction time (0–100)	54.4 (9.3)	57.5 (10.5)	3.5 (7.4)	0.001	54.6 (11.8)	54.2 (9.4)	−0.3 (7.0)	0.60	0.53	0.039
Detectability (0–100)	51.6 (9.7)	46.6 (11.2)	−5.5 (7.9)	<0.001	53.9 (9.2)	53.0 (9.6)	−1.0 (7.7)	0.11	0.57	0.004
**Sleep disturbance**										
**Pittsburgh Sleep Quality Index (PSQI)**										
Use of sleeping medication (0–3)	1.0 (1.4)	0.9 (1.4)	−0.1 (0.9)	0.51	1.0 (1.4)	1.0 (1.4)	−0.1 (1.1)	0.44	0.03	0.94
PSQI global score (0–21)	9.7 (4.6)	8.1 (3.9)	−1.5 (3.1)	0.002	10.6 (5.1)	10.0 (4.8)	−0.5 (3.6)	0.54	0.31	0.06
**Psychological distress**										
**Brief Symptom Inventory**										
Global Severity Index (0–4)	0.8 (0.5)	0.6 (0.6)	−0.3 (0.5)	<0.001	1.0 (0.7)	0.8 (0.7)	−0.2 (0.4)	<0.001	0.18	0.17

*p-Values indicate statistically significant differences within (p_within_) and between (p_between_) groups*.

*Cohen’s d describes the size of differences between groups*.

*^a^Standardized T-scores*.

### Yoga Classes

To ensure that yoga classes were carried out uniformly across the different correctional facilities, there were specific guidelines for the classes formulated by Bilderbeck et al. (20) in cooperation with the Prison Phoenix Trust. Yoga classes were held once a week, lasted for 90 min, and comprised a combination of *asanas* (yoga postures), breathing exercises, deep relaxation, and meditation. Each yoga class contained the following:

- Exercises to warm up and increase the blood circulation in the body, minimum 10 min.
- Sixteen specific postures, such as Boat Pose, Downward-Facing Dog Pose, and Tree Pose.
- At least two of the more powerful postures, such as Triangle Pose, Warrior II Pose, or Extended Side-Angle Pose.
- One or two breathing exercises, minimum 5 min.
- Corpse Pose for relaxation at the end of the yoga class, minimum 5 min. The final part of the yoga class often included *Yoga Nidra*, which is an exercise for deep relaxation.

Guidelines also included information on how the yoga teachers could support participants and adapt or simplify postures and exercises to meet participants’ special needs.

### Measures

At pre- and post-intervention, participants completed a number of questionnaires and a computerized test measuring attention and impulsivity. The pre-intervention assessment also included questions on education, medical background, medications, and expectations of yoga. The post-intervention assessment included questions on medications, participation in treatment programs, time spent on yoga and physical activity, and how the treatment group experienced the yoga. Measures administered in this study were based on Bilderbeck et al. study (20), with some exceptions. Some instruments were added [such as the Prison Adjusted Measure of Aggression (PAMA) and the Pittsburgh Sleep Quality Index (PSQI), described in detail below], an extended version of the affective state measure [Positive and Negative Affect Schedule-Expanded form 30 items (PANAS-X30), description please see below] was administered and a more complex instrument to measure attention and impulsivity [Continuous Performance Test II (CPT II), description please see below] was used in our study. All measurements referred back to the last month.

#### Perceived Stress Scale—14 Items (PSS-14)

The PSS-14 (24) is a 14-item self-report inventory used to measure the degree to which situations in one’s life are perceived as stressful. The validity and reliability of the Swedish translation of PSS-14 has not yet been tested, but the English version is a valid, reliable measure of perceived stress [such as internal consistency, Cronbach’s alpha 0.84–0.86 in three different samples (24)].

#### Prison Adjusted Measure of Aggression (PAMA)

Prison Adjusted Measure of Aggression is an 11-item instrument used to measure the frequency of prison inmates’ aggressive and antisocial behavior during the past month. It is an adapted version of the Life History of Aggression scale [LHA (25)]. Adaptations were made by the Forensic Psychiatry Group at the Sahlgrenska Academy and included the time frame and wordings of certain items. Whereas the LHA measures aggressive and antisocial behavior since age 13 (trait aggression), PAMA focuses on past month’s aggressive and antisocial behavior (and has the capacity to detect changes in aggressive behavior). The format of PAMA remains the same as that for LHA, with 11 items distributed over three subscales. These subscales are as follows: (1) a 5-item aggression subscale (which measures overt aggression and includes items on temper tantrums, verbal and indirect aggression, nonspecific fighting, and physical assault against people), (2) a 2-item, self-directed aggression subscale (which includes items on self-injurious behavior and suicide attempts), and (3) a 4-item antisocial behavior subscale (which measures antisocial behavior and its consequences with items on disciplinary problems in the correctional facility, problems with supervisors, and antisocial behavior resulting in or not resulting in correctional officers involvement) (26). Items are rated on a 6-point scale based on the total amount of occurrences during the last month: 0 = no occurrences, 1 = 1 event, 2 = 2 or 3 events, 3 = 4–9 events, 4 = 10 or more events, and 5 = more events than can be counted. In a validation study, the psychometric properties of PAMA were examined, where the subscales and the total scale showed an acceptable level of internal consistency [Cronbach’s alphas between 0.67 and 0.97; (26)].

#### Positive and Negative Affect Schedule—Expanded Form 30 Items (PANAS-X30)

PANAS (27) is an instrument used to measure two general, affective state dimensions: positive and negative affect. It consists of 20 items, evenly distributed over the 2 dimensions (27). PANAS has demonstrated good internal consistency (Cronbach’s alpha, 0.86–0.90 for the positive affect dimension and 0.84–0.87 for the negative affect dimension), good test–retest reliability, and excellent convergent and discriminant validity (27). To cover a broader spectrum of affects, researchers have emphasized the importance of considering both valence (positive/negative) and activation (28, 29). On this basis, two dimensions (each consisting of five items) were added to measure positive and negative affects that are low in activation. The PANAS questionnaire used in this study (PANAS-X30) consisted of 30 items distributed over four dimensions: positive, respectively, negative activated affects (the positive and negative affect dimensions in PANAS) and positive, respectively, negative deactivated affects (the extra items described above). The validity and reliability of the latter dimensions have not been tested before, but showed good internal consistency at both pre- and post-intervention assessment in this study (Cronbach’s alpha, positive deactivated affects: 0.80 and 0.88; negative deactivated affects: 0.77 and 0.85).

#### Conners’ Continuous Performance Test II (CPT II)

Conners’ CPT II (30) is a computerized test that measures attention, impulsivity, and vigilance. There is little or no practice effect (31), which makes the test suitable for repeated measurements. During the test, 360 letters are shown at varying intervals. The respondent is required to press the space bar when any letter *except* the letter “X” appears on the screen. The respondent is instructed to respond as fast, but also as accurately as possible. Post-administration, a profile report containing the respondent’s performance in *T*-scores on a number of variables is generated. This study focused on four of the variables: omissions (targets to which the respondent did not respond), commissions (responses to non-targets), hit reaction time (mean response time for all target responses), and detectability (how well the respondent discriminated between targets and non-targets). The four variables were chosen because they measure different aspects of attention and impulsivity and have better test–retest reliability than other variables in Conners’ CPT II [*r* = 0.55–0.84; (31)].

#### Pittsburgh Sleep Quality Index (PSQI)

PSQI (32) is a self-report inventory used to assess sleep quality and sleep disturbances over the previous month. The PSQI consists of 19 items generating seven weighted component scores and one global score. The present study focused on the PSQI global score and the component score use of sleeping medication. The PSQI has shown good internal consistency (overall Cronbach’s alpha for all seven components: 0.83), stability across time, and ability to discriminate patients with sleeping disturbances from controls without sleep complaints (32).

#### Brief Symptom Inventory (BSI)

BSI (33) is a self-report inventory used to measure psychological distress and psychiatric symptoms. The inventory is a brief version of the Symptom Checklist-90-Revised. It consists of 53 items, distributed over 9 primary symptom dimensions or constructs. Researchers using the BSI have generally concentrated on the Global Severity Index (GSI). The GSI is a weighted frequency score, based on the sum of ratings on all 53 items. It is the single best indicator of current distress (33), so it was used as a global measure of psychological distress in this study.

### Statistical Analyses

Since few female prison inmates participated in the study (*n* = 19, post-attrition), male and female participants could not be analyzed separately. No intra-individual analyses could be made for the control group participants who completed all three assessments, since these were low in number (*n* = 22–24 on the different measures at Time 3 assessment). Due to data violating the assumption of normality, all statistical tests were non-parametric. All statistical analyses were conducted using a significance level of *p* < 0.05. If nothing else is specified, continuous variables are presented by mean (M) and standard deviation (SD) and categorical variables by number (*n*) and percentage (%). Only complete post-attrition data were analyzed (per protocol analysis), so the number of analyzed cases varied on the different measures.

Comparison of attrition rate between the groups was calculated with Fisher’s exact test. Comparisons of Time 1 to Time 2 scores within groups were performed using the Wilcoxon signed-rank test. Mean change and SDs were calculated for differences within groups. Mann–Whitney *U* tests were used for continuous variables and Fisher’s exact test for categorical variables to compare differences between groups. Effect sizes (Cohen’s *d*) were calculated for differences between groups. An effect size of 0.2 was considered a small effect, 0.5 a medium effect, and 0.8 a large effect (34).

Bivariate Spearman rank order correlation analyses were conducted between yoga participants’ expectations of the effect of yoga and the outcome on selected variables to control for subject-expectancy effects: total score on PSS-14; the aggression subscale in PAMA; the positive deactivation and positive activation dimensions in PANAS; and the PSQI global score. These variables were selected because they match the expectations in the pre-intervention assessment (i.e., stress, anger/frustration, calm, happiness, and sleep).

### Ethical Considerations

The study was approved by the regional Ethical Review Board in Linköping (2013/302-31). Prison inmates interested in participating received both verbal and written information about the study procedure and conditions of participation. Written informed consent was obtained from all participants. Upon study completion, participants received a phone card valued at 200 SEK. Control group participants who completed the Time 3 assessment were given a yoga gift valued at 200 SEK.

## Results

When Time 1 and Time 2 scores on the different variables were compared within and between the yoga and control groups, several significant differences were found.

Within the yoga group, there were significant differences (improvements) on 13 of the 16 variables (e.g., less perceived stress, better sleep quality, an increased psychological and emotional well-being, less aggressive, antisocial, and self-harm behaviors) (Table 3). Within the control group, there were significant differences on two (less perceived stress and less aggressive behavior) of the 16 variables (Table 3).

Between groups, six statistically significant differences were revealed on the following variables: antisocial behavior, positive deactivated affect, negative activated affect, commissions, hit reaction time, and detectability. Effect size Cohen’s *d*, on the significant between-groups differences, ranged from 0.29 to 0.72. Table 3 presents results from the analyses within and between groups on all variables.

### Perceived Stress

No significant difference was found between the yoga and control groups on the variable that measured perceived stress, due to the fact that both groups showed a significant decrease in perceived stress at Time 2 compared to Time 1. The average change between Time 1 and Time 2 was slightly greater in the yoga group compared to the control group (−3.8 compared to −1.7) but was not statistically significant.

### Aggression

**Figure 2:**
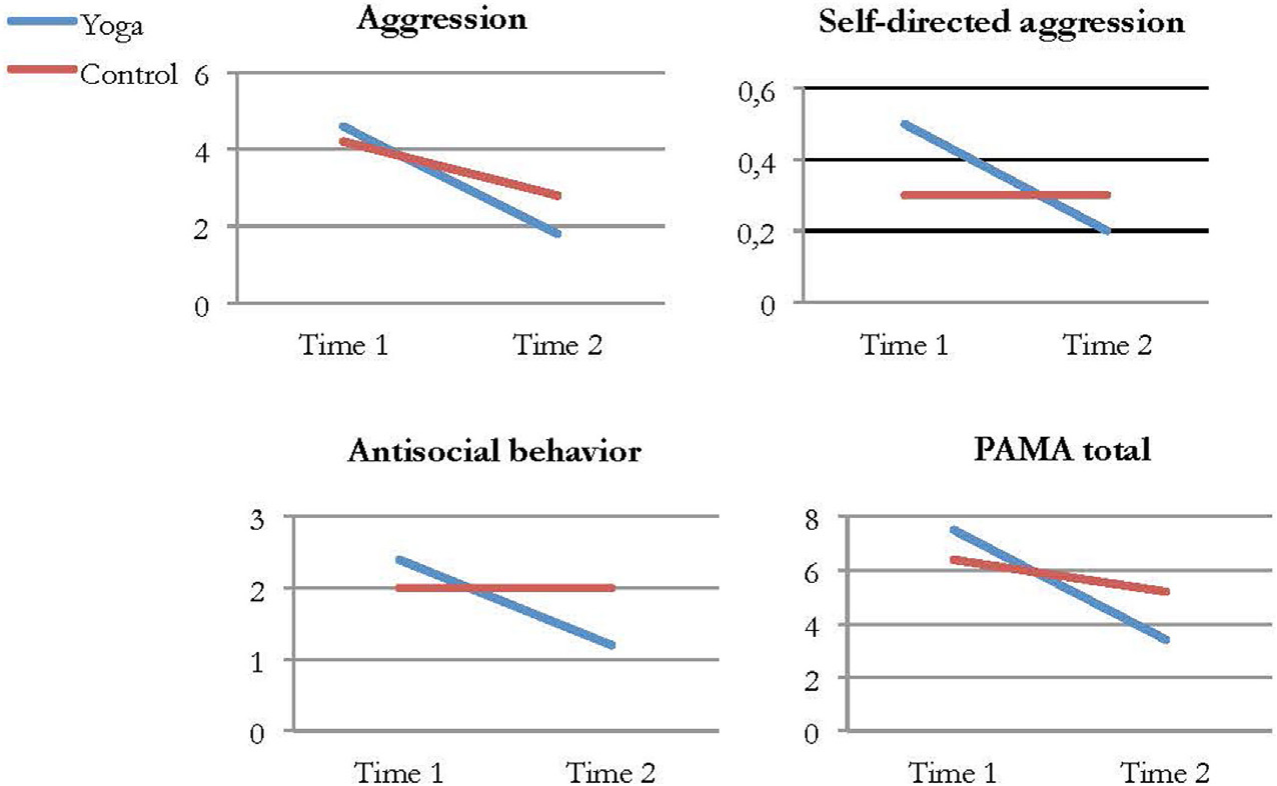
Average ratings of aggression, self-directed aggression, antisocial behavior, and total score on the Prison Adjusted Measure of Aggression (PAMA) in the yoga and control group at Time 1 and Time 2.

Aggression was measured using the self-report instrument PAMA, which consists of three subscales and a total scale. One significant difference between the yoga and control groups was found on the subscale that measured antisocial behavior. Compared to the control group, yoga group participants showed a significant decrease in antisocial behavior from pre- to post-intervention. The effect size was close to medium, at *d* = 0.45. Analyses within groups showed significant improvements in the yoga group on all subscales and the total scale, whereas there were no significant differences in the control group. Figure 2 shows yoga and control group participants’ average ratings on the PAMA subscales and total scale at Time 1 and Time 2.

### Positive and Negative Affect

**Figure 3:**
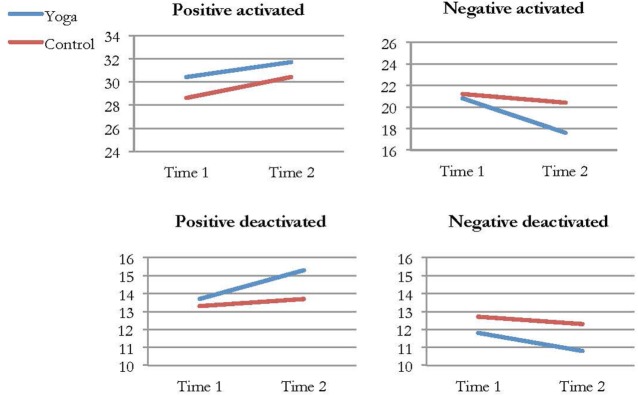
Average ratings of positive and negative activated and deactivated affect in the yoga and control group at Time 1 and Time 2.

Positive and negative affect was measured using the self-report instrument PANAS-X30, which consists of four dimensions. On two of the dimensions, significant differences between the yoga and control groups were found. From pre- to post-intervention, the yoga group reported a significant increase of positive deactivated affect and a significant decrease of negative activated affect compared to the control group. In both cases, the effect size was small, *d* = 0.29 and *d* = 0.33, respectively. Analyses within groups revealed significant improvements in the yoga group on the positive deactivated affect, negative activated affect, and negative deactivated affect dimensions. No significant differences regarding positive or negative affect were found within the control group. Figure 3 shows yoga and control group’s participant’s average ratings on the four different dimensions in PANAS-X30 at Time 1 and Time 2.

### Attention and Impulse Control

**Figure 4:**
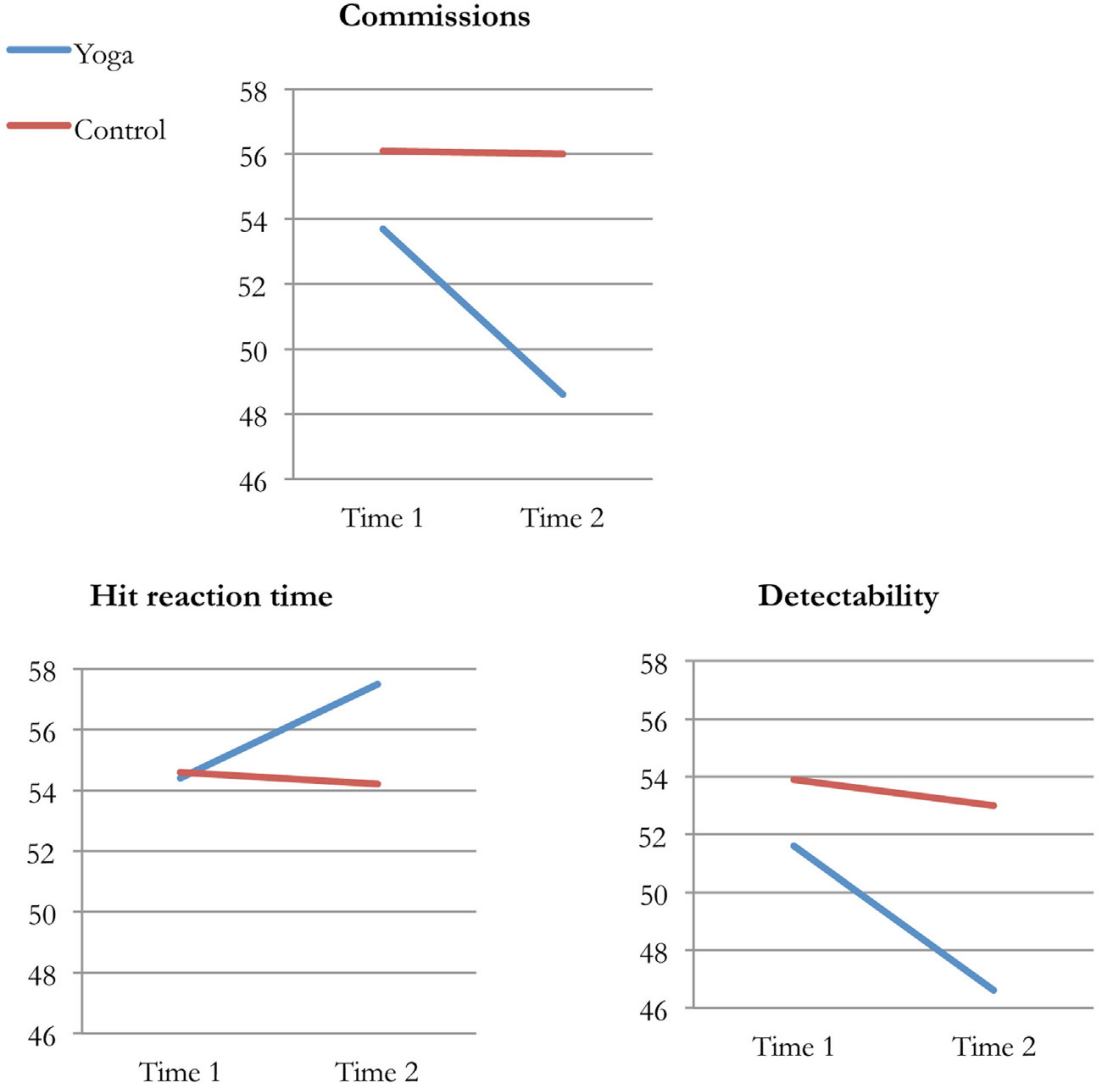
Average performance (*T*-scores) on commissions, hit reaction time, and detectability in the yoga and control group at Time 1 and Time 2.

Four of the variables in the Conners’ CPT II profile report were selected to measure attention and impulse control. Significant differences between the yoga and control group were found on three variables. From pre- to post-intervention, the yoga group had significantly fewer errors of commission, increased hit reaction time, and better detectability compared to the control group. Effect sizes were medium or close to large: *d* = 0.72, *d* = 0.53, and *d* = 0.57. Analyses within groups showed significant differences from pre- to post-intervention in the yoga group on the same three variables as above. No significant differences were found in the control group. Figure 4 shows yoga and control groups’ average performances on commissions, hit reaction time, and detectability at Time 1 and Time 2.

### Sleep Quality

No significant differences between the yoga and control groups were found on the variables PSQI global score and use of sleeping medication. However, there was tendency on the PSQI global score, where the *p-*value between groups was close to significant, *p* = 0.06. Here, the yoga group had a slightly greater average decrease in scores from pre- to post-intervention than the control group (−1.5 compared to −0.5). Decreasing scores on the PSQI indicate improvements in sleep quality. Analyses within groups showed significant improvements in global sleep quality in the yoga group, but not in the control group. No differences were found in any of the groups in regard to use of sleeping medication.

### Psychological Well-being

No significant difference between groups was found on the variable that measured psychological well-being. Analyses within groups showed that both groups had significantly lower scores on the general severity index at Time 2 compared to Time 1, which indicates improvements in psychological well-being.

### Expectations

No correlations were found between yoga participants’ expectations of yoga and the outcome on selected variables. All correlation coefficients were weak, from −0.16 to 0.13, which indicates that expectations of yoga did not affect yoga participants’ self-reports.

## Discussion

Within the yoga group, improvements from pre- to post-intervention were found on almost all analyzed variables. When improvements in the yoga group were compared to those of the control group, some significant differences between groups were revealed. Relative to the control group, participants in the yoga group showed increased positive affect, impulse control and attention, and decreased negative affect and antisocial behavior.

Results show that Krimyoga is associated with improved emotional well-being. Positive affective states, such as calm, relaxed, and content, increased, while negative affective states, such as hostile, upset, and nervous, decreased. These results differ from those of Bilderbeck et al. (20), in which improvement in the yoga group was only found on the dimension that measured positive activated affect. Differences in results were partly due to variations in the PANAS instruments. Bilderbeck and colleagues administered the original 20-item PANAS, while our study used an extended version. Results from this study and Bilderbeck et al. (20) suggest that yoga, in comparison to other physical activities, has a specific, positive effect on prison inmates’ emotional well-being.

One of the most interesting findings for the Swedish Prison and Probation Services should be the significant decrease in antisocial behavior in the yoga group. The subscale that measures antisocial behavior consists of items on antisocial acts (such as school disciplinary problems, problems with supervisors, or conflicts with other people) and their consequences (such as, warnings, reports, and solitary confinement). After 10 weeks of yoga, yoga participants reported a significant decrease of antisocial behavior, compared to the control group. Since antisocial personality pattern is one of the criminogenic needs (35), the association between yoga and decreased antisocial behavior is highly interesting.

The results on Conners’ CPT II stand out the most. After 10 weeks of yoga, inmates’ performance had improved significantly on three of four variables (commissions, hit reaction time, and detectability), in comparison to the control group. An increase in hit reaction time at Time 2 could be interpreted as a decline, since the test person is instructed to respond as fast as possible. However, yoga participants made fewer errors of commission and were more accurate in discriminating between targets and non-targets at Time 2. It is likely that the longer hit reaction time resulted in more accurate responses. Accordingly, the increase in hit reaction time can be interpreted as positive. The results on Conners’ CPT II are interesting in many ways. First, the computerized test measures behavior in a way that is relatively objective, which means that results are solid. Second, effect sizes were mostly medium, with one being close to large. In their 2015 meta-analysis, Auty and colleagues concluded that a larger effect size was demonstrated for psychological well-being than for behavioral functioning. Our study showed the contrary: the largest effect sizes were found on the computerized test that measured attention and impulse control. Finally, it is possible that improvements in attention and impulse control in the yoga group were associated with the reduction in antisocial behavior, since impairments in executive functions have been linked to antisocial behavior (36, 37).

Prior to this study, no yoga study in correctional settings had explored the effect of yoga on sleep. Quality of sleep was included as a variable in the present study because it is strongly associated with physical and psychological well-being, as well as psychosocial functioning. In previous research, poor sleep quality has been found to relate to higher levels of aggressiveness and hostility in prison inmate populations (38, 39). In the present study, no significant difference was found between the yoga and control groups on any of the two variables that were used to measure sleep quality. However, there was a tendency on the variable PSQI global score for the yoga group to have a slightly greater improvement in overall sleep quality from pre- to post-intervention, compared to the control group. If more participants had completed the PSQI and obtained a global score, a level of statistical significance between the two groups might have been reached on this variable.

On the variable of perceived stress, no significant difference between groups was found, despite the fact that Bilderbeck et al. (20, 21) found significant improvements in stress in the yoga group. Instead, both groups in this study reported a significant decrease in perceived stress at Time 2 compared to Time 1. One possible explanation for the significant improvements in the control group is that participants were required to engage in a physical activity for 90 min each week during the study period. Studies have found associations between physical activity and lower levels of stress (40). Accordingly, the physical exercise in which the control group participants engaged could have had positive effects on their perceived stress levels. However, it should be mentioned that the control group in Bilderbeck et al.’s (20) study also engaged in physical activity, but a significant difference between the yoga and control groups was still found on the variable of perceived stress. The post-intervention assessment in our study included a question on time spent on physical activity. The answers indicated that 79.5% of the control group engaged in more than 2 h physical activity every week during the study. This may explain the significant improvement of perceived stress level in the control group in our study.

This study shows evidence that 10 weeks of yoga in correctional settings improves inmates’ emotional well-being and has positive effects on criminogenic needs, such as impulsivity and antisocial behavior. When practiced in correctional facilities, yoga can counteract deteriorating effects of imprisonment on mental health and contribute to an improved well-being. Also, yoga can function as a pro-social leisure activity that can help re-integration of prison inmates upon release into society, which can possibly reduce the risks of recidivism.

### Strengths and Limitations

Randomized controlled trials are generally considered the gold standard for clinical trials, because this type of design reduces the risk for biases. Therefore, the design in this study is a strength. It is likely that prison inmates who chose to participate were interested in yoga and possibly believed in its positive effects, which could have affected the outcome. However, we tested for subject-expectancy’s effect and there was no indication of correlations between yoga participants’ expectations and the outcome on selected variables.

Still, there are limitations in the study that must be acknowledged. One of them concerns attrition. Participant attrition in this study was large, but not larger than in previous yoga studies in correctional settings; Bilderbeck et al. (20) reported an attrition rate of about 40%, whereas Harner et al. (41) reported an attrition rate of just above 70%. However, certain differences were found between the group of participants lost to follow-up and the group that completed our study. Participants lost to follow-up were significantly younger than those who completed the study and had higher ratings on the PAMA subscales that measure aggression and antisocial behavior at Time 1. This indicates that younger inmates with a higher level of externalizing behaviors had difficulties completing the study. Similar tendencies were found in Bilderbeck et al.’s (21) study showing that yoga class attendance was positively correlated with age of participants. In addition to the age difference of dropouts in the present study, the yoga and control groups differed in attrition rates and reasons for attrition.

Moreover, nobody verified that the control group engaged in physical activity for 90 min each week, neither was the control group asked to keep an exercise diary. However, post-intervention assessment suggests that the majority of the control group (91.8%) engaged in at least one h of physical activity every week during the study. Furthermore, the format of the physical activity and yoga differed. Unlike the unstructured physical activity in which the control group participants engaged, yoga was practiced in a group. Receiving attention from the yoga teacher and belonging to a group are factors that could have contributed to the positive effects in the yoga group. However, based on research that compared yoga to physical group activities (42), it is unlikely that the positive effects of yoga found in this study were due to the group setting.

The role of the yoga teachers in this study can be viewed as a strength as well as a limitation. Since the yoga teachers at the correctional facilities were the ones collecting data and holding yoga classes, this study was less standardized than previous studies that usually had external yoga teachers and researchers that conducted all assessments. However, the main aim with this study was to evaluate the current yoga practice in Swedish correctional facilities, which could not have been achieved with external yoga teachers running the classes. Also, because of the many correctional facilities that participated in the study, and their geographical locations, having researchers conducting all assessments was not a viable option.

Finally, the statistical analyses are associated with certain limitations. Due to multiple tests, there was an increased risk for type 1 errors. Also, due to attrition and missing data, there was limited statistical power on some of the variables, which meant an increased risk for type 2 errors.

## Author Contributions

NK was the project leader. She has planned the study and actively participated in the analytical work and the manuscript writing. CF and SA were project coordinators. Beside the everyday work of a project coordinator, CF participated in the planning and the initiation of the study and reviewed the manuscript, while SA actively participated in the analytical work and the writing of the manuscript.

## Conflict of Interest Statement

The authors during the study were employed of the Swedish Prison and Probation Services.
